# A Chapter on Cholera for Lay Readers

**Published:** 1893-09

**Authors:** 


A CHAPTER ON CHOLERA FOR LAY READERS: HISTORY, SYMPTOMS, PREVENTION, AND TREATMENT OF THE DISEASE. By Walter Vought, Ph. B., M. D., Medical Director and Physician-in-Charge of the Fire Island Quarantine Station, Port of New York; Fellow of the New York Academy of Medicine, etc. Illustrated with colored plates and wood engravings. In one small 12mo volume, 110 pages. Price, 75 cents net. Philadelphia: The F. A. Davis Co., Publishers, 1914 and 1916 Cherry Street.

This is a neat little volume which is quite seasonable, now that every one is expecting an outbreak of this dread scourge. It is an excellent little book, giving in small compass the history of cholera, its causes, character, symptoms, diagnosis, prognosis, treatment, prevention, quarantine, and disinfection. All these points are ably discussed, and the latest information given. It is, however, in the matter of treatment where the little volume seems deficient, even from the old-school standpoint. The treatment most highly recommended is Calomel, in doses of from one-third to three-fourths grain, every two or three hours. The pains are to be relieved by hypodermic injections of Morphine, and vomiting is relieved by champagne or brandy and water.

Generally speaking, the little volume is a welcome addition to the rapidly accumulating literature of cholera. It is a good book for circulation among the laity provided they do not attempt to treat themselves without the aid of a physician.

